# Practical Considerations in Abdominal MRI: Sequences, Patient Preparation, and Clinical Applications

**DOI:** 10.3390/mps8060129

**Published:** 2025-11-01

**Authors:** Nicoleta Cazacu, Claudia G. Chilom, Cosmin Adrian, Costin A. Minoiu

**Affiliations:** 1 Department of Radiology, Smeeni Chronic Disease Hospital, 127595 Buzău, Romania; 2 Department of Electricity, Solid Physics and Biophysics, Faculty of Physics, University of Bucharest, Str. Atomistilor No. 405, CP MG-11, Bucuresti-Magurele, 077125 Magurele, Romania; 3 Department of Radiology, Clinical Emergency Hospital Bucharest, 014461 Bucharest, Romania; 4 Department of Radiology, Carol Davila University of Medicine and Pharmacy, 050474 Bucharest, Romania

**Keywords:** MRI, upper abdomen, sequences, clinical application, hepatic lesions

## Abstract

This study discusses the challenges encountered in implementing a detailed protocol for upper abdominal imaging using magnetic resonance imaging (MRI), ranging from patient preparation and sequence selection to clinical applications. MRI is a valuable non-invasive imaging modality employed both in the early detection of diseases and as a complementary tool for the detailed characterization of various pathologies. Nevertheless, performing an abdominal MRI examination can be challenging; therefore, the understanding of sequences is particularly important, as changing the parameters can not only influence the quality of the images but also optimize scanning time improve patient experience during the examination. The methodology illustrates the purpose of each sequence and the critical role of appropriate patient preparation. Results highlighted the significance of these factors in the evaluation of hepatic lesions, showing that the proper choice of sequences and parameters is essential for distinguishing benign from malignant findings and for achieving an accurate diagnosis. It was also shown that MRI plays an important role as a complementary technique in investigation of upper abdominal pathologies in order to avoid overexposure to radiation.

## 1. Introduction

In recent years, there has been much debate concerning the positive aspects of using MR imaging and whether this method is safe or not. Abdominal MRI is a technique that has been increasingly popular in recent years due to its high sensitivity to detect and characterize lesions of abdominal organs, as well as to monitor the response to treatment or postoperative outcomes [1].

Abdominal MRI can also be used as a complementary technique for a more complex characterization of pathologies. Noone et al., 2004 [2] showed that, among the common methods used to investigate abdominal organs, MRI provides the most accurate diagnoses in the investigation of hepatic, adrenal, and pancreatic disease. In comparison, the use of ultrasound provides the most accurate diagnoses in the investigation of gallbladder disease, but CT or MRI may be more appropriate for the detection of renal disease [2].

The result obtained using ultrasound can be affected by artifacts related to the patient, equipment, or operator (e.g., shadowing, reverberation, main bang artifact) [3]. Despite continuous technological progress, these limitations persist and may reduce diagnostic accuracy. A combined diagnostic strategy using ultrasound and MRI for the upper abdomen may lead to improved specificity of diagnosis compared to ultrasound alone [4].

**Scheme 1:**
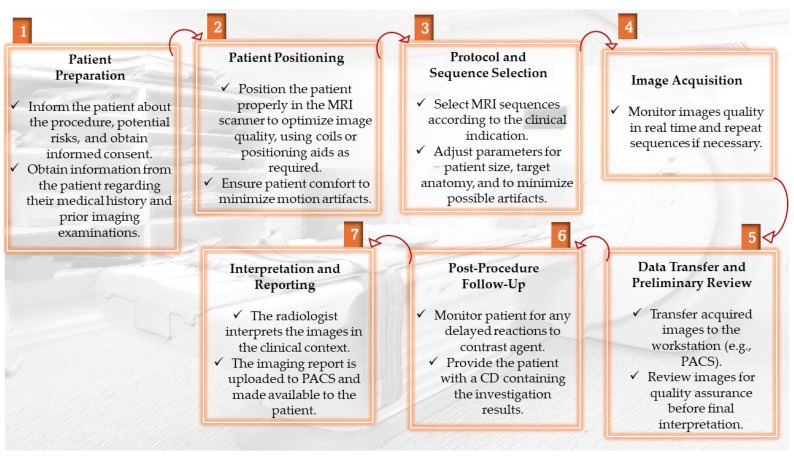
Workflow of the upper abdominal MRI examination. This includes patient scheduling and screening for MRI contraindications, collection of medical history, accurate positioning within the scanner, selection of the protocol, real-time monitoring of image acquisition, and post-scan verification of the patient. All these steps contribute to obtaining diagnostically interpretable images while ensuring patient safety.

Scheme 1 presents an overview of the upper abdominal MRI workflow, from initial patient preparation to the finalization of the examination.

From Step 1, Patient Preparation, to Step 7, Interpretation and Reporting, every stage is crucial for the success of an MRI examination. However, since the duration of the MRI investigation is usually quite long, image quality can be affected by motion artifacts or the patient’s inability to hold their breath properly. Thus, important anatomical structures and lesions may be obscured. Furthermore, patients find it uncomfortable to be still and enclosed in the scanner for extended periods of time. Lee et al., 2000 [5] and Krinsky et al., 2001 [6] reported that 7% of patients undergoing hepatic MRI were unable to hold their breath for 15 s [7]. Even if MRI is a highly recommended technique in the abdominal evaluation of pathologies, due to its capacity to provide multiparametric images for lesions, many patients cannot benefit from this investigation due to claustrophobia or implants incompatible with MRI. In these cases, communication with patients during MRI examinations, facilitated by the MRI audio system used between sequences, may help reduce discomfort and anxiety, particularly in claustrophobic patients, thus increasing their sense of safety and cooperation. Moreover, continuous communication is essential in patients with implants, even when official documentation of MR compatibility is available, to promptly detect any discomfort or heating sensations and to ensure safety throughout the examination.

**Scheme 2:**
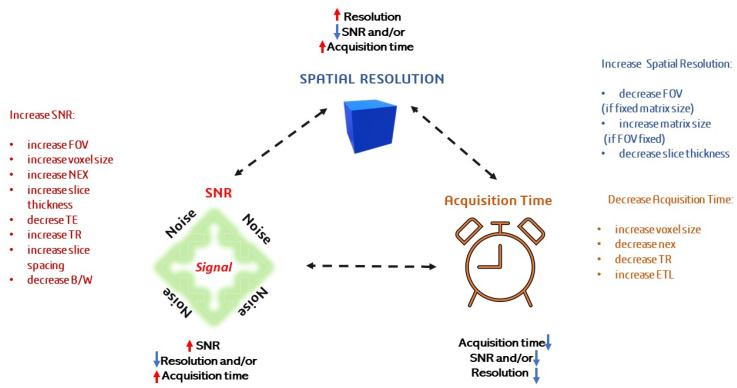
Trade-off parameters in MRI. Increasing NEX and repetition time (TR) enhances SNR while also increasing scan time. As a result, finding the appropriate acquisition parameters to optimize SNR within a given total acquisition time is essential. Decreasing the matrix size while keeping the FOV constant leads to improvements in spatial resolution but at the cost of reduced SNR and increased acquisition time. The operator must prioritize the most relevant imaging factor based on the clinical context and patient’s condition.

The longer acquisition time often causes MRI to fail to capture the optimal phase during dynamic phase acquisition, as the arterial window is relatively short [8,9]. The arterial phase is a post-contrast period that occurs 15–25 s after contrast injection. Images in this phase show the hepatic artery and branches fully enhanced, but the hepatic veins may not yet be enhanced by antegrade flow. Recent studies indicated that double or even triple-arterial phase dynamic MRI demonstrated higher sensitivity for hepatic lesions compared to single arterial phase [10,11]. However, interpretation of these studies is challenging as the definition of arterial phase is based on a fixed time delay and not determined based on current definitions as provided by the Liver Imaging Reporting and Data System (LI-RADS v2014) [10,11]. Changing some acquisition parameters can reduce scan time, but this comes at the cost of some aspects of image quality like spatial resolution. Also, respiratory triggering and navigation are nowadays routinely used for free-breath volume scanning to mitigate respiratory artifacts, but with relatively long acquisition time, while breath-hold imaging is a speedier approach, but it can result in a lot of artifacts for patients who cannot hold their breath. MRI investigation involves a trade-off between (1) spatial resolution, which is restricted by voxel size (decided by matrix size, field of view, and slice thickness), (2) signal-to-noise ratio (SNR), dictated by voxel volume, square root of the number of averages (NSA or NEX), and receiver bandwidth, and (3) total scan time (Scheme 2). Parameter adjustments must be performed with care, as they can significantly affect image quality. For example, inappropriate modification of the repetition time (TR) or echo time (TE) can alter the type of contrast. While T2-weighted images typically require longer TR and TE values (e.g., approximately TR > 2000 ms; TE = 90–140 ms), T1-weighted images generally use shorter values (approximately TR = 300–600 ms; TE = 10–30 ms) [12].

Regarding NEX, it is important to note that SNR increases proportionally to the square root of NEX, while acquisition time increases linearly. For example, with NEX = 1, the acquisition time may be 2.42 min and SNR at baseline (1); increasing NEX to 2 nearly doubles the acquisition time to approximately 5 min and increases SNR by a factor of √2 (~1.41) [13]. Optimizing NEX requires balancing image quality with practical constraints such as scan duration and patient tolerance. Increasing the matrix size while keeping the FOV constant leads to a higher number of pixels. Since the FOV remains fixed, the individual pixels become smaller, resulting in improved spatial resolution but at the cost of reduced signal-to-noise ratio (SNR) and increased acquisition time [13]. Another parameter that can be adjusted by the operator to improve MRI image quality is the receiver bandwidth, which represents the range of frequencies sampled by the readout gradient. A narrow bandwidth reduces image noise but may introduce image blurring or distortion, while a broad bandwidth increases noise yet improves tissue contrast and reduces susceptibility artifacts [14].

**Scheme 3:**
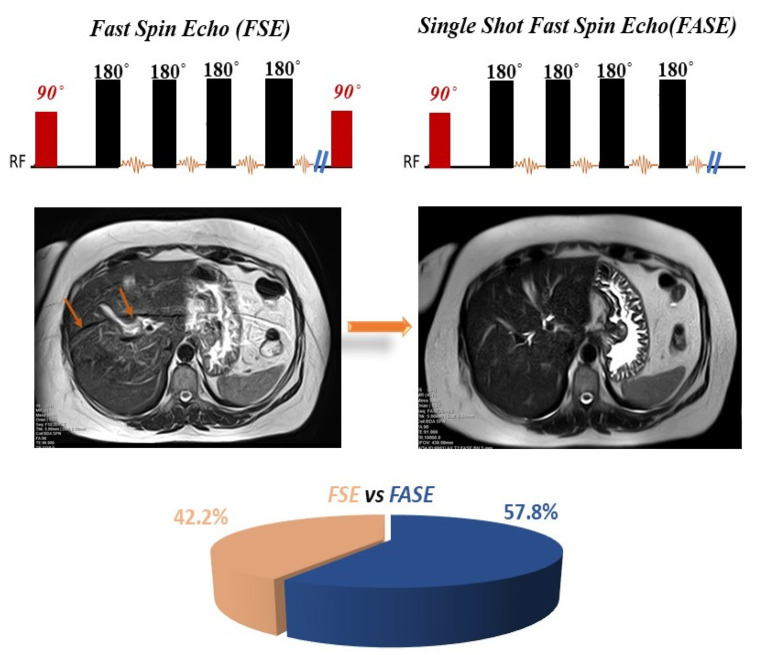
Comparison of sequences that can be used in abdominal MRI. Left—FSE in abdominal analysis with respiratory artifacts (orange arrow). Right—the same examination but with FASE, the artifacts were removed.

The MRI sequences used in this study are well established and have been previously described in the literature [15,16,17,18,19,20,21,22,23,24,25], ensuring reproducibility and comparability with others research. It is important to mention that the protocol applied may vary depending on the patient’s pathology and is established together with the radiologist. Also, the parameters of each sequence can be easily modified when artifacts appear, such as metallic artifacts, respiratory artifacts (Scheme 3), aliasing, and others. The main sequences used in this study are presented as follows:

- Single-Shot Fast Spin Echo (Canon: FASE) is a spin echo sequence, which uses the single-shot technique to acquire the data after a single 90˚ radio frequency (RF) excitation pulse, followed by a series of 180˚ RF pulses. All the data (e.g., 256) are acquired in a single TR (Scheme 3—right); therefore, all the lines from the k space are completed at the same time. In the case of Fast Spin Echo (FSE), a multi-shot technique, the number of shots depends on the value of echo train length (if echo train length (ETL) = 16 and Phase Encode Matrix Size = 256, then the number of shots is equals to 256 divided by 16) (Scheme 3—left). Using FASE, sequences with a shorter duration are obtained, which, in the case of the abominable MRI, leads to the reduction or even elimination of movement artifacts (Scheme 3). A total of 45 abdominal examinations were evaluated to assess the feasibility of performing FASE versus FSE sequences. These data were used to provide a statistical overview of sequence applicability. Detailed morphological analysis is illustrated through representative cases, rather than for all patients individually. Out of the 45 patients from the Smeeni Chronic Disease Hospital, only 19 (42.2%) were able to adequately hold their breath during the FSE sequence. For the 26 patients (57.8%), the faster FASE sequence was required, as it allowed for a shorter acquisition time, reducing the challenges associated with breath-holding (Scheme 3).

- Steady-State Free Precession is a gradient-echo sequence used in different applications like cardiac imaging, great vessels, abdominal imaging, cervical spine, and internal auditory meatus (IAM), as well as in other investigations because the cerebrospinal fluid (CSF) flow is reduced [15]. This technique is especially useful for depicting tissue and blood vessels with relatively long T2 during breath-holding, and it presents a great interest due to inherently high SNR and a very short acquisition time. The contrast obtained is more T1/T2 than T2 or T1, which is useful for a good contrast between fluids and surrounding tissue, allowing visualization of vascular structures and the distribution of body fluids with great contrast-to-noise ratio (CNR). An important aspect is that this sequence is not used to visualize diverse lesions because the difference between normal tissue T2/T1 ratio and lesion T2/T1 ratio might be quite small [16]; it is also susceptible to artifacts and have loud gradient noise.
- In Spin Echo–Echo Planer Imaging (SE-EPI)*,* the RF excitation pulse is followed by a single refocusing pulse, similar to the Spin Echo sequence [17], but then multiple echoes are generated using the reversed polarity of the readout gradient and acquired using phase-encode gradient pulses of varying sizes. This technique uses Half-Fourier Acquisition, or Asymmetric Fourier Imaging (AFI), in the phase-encode direction in order to reduce TE (and increase the echo factor).

In addition to rapid sequences such as HASTE and FASE, acquisition strategies and advanced radial k-space–based techniques, such as GRASP (Golden-angle RAdial Sparse Parallel) and DISCO (DIfferential Subsampling with Cartesian Ordering), play important roles in improving image quality, motion robustness, and temporal resolution.

In order to obtain an optimal MRI image, state-of-the-art MRI systems have been implemented, and applications have been developed to deal with pulse sequences, image reconstruction, and data analysis [18]. Deep learning (DL) technology already plays an important role in the analysis and post-processing of the images, and even in the image acquisition and image reconstruction itself, which makes it one of the most important upcoming developments in radiology [19].

## 2. Materials and Methods

### 2.1. Patient Preparation

All the patients underwent upper abdominal MRI examinations with intravenous contrast enhancement. Before the examination, each patient was screened for contraindications and instructed about the procedure. For each patient, laboratory analyses were evaluated to ensure the safe administration of contrast agents without adverse effects on renal function. Positioning was performed in the supine position with appropriate coil placement, and a respiratory sensor was positioned on the diaphragm to monitor and verify patients’ compliance with breathing instructions during abdominal MRI sequences. Patients were informed about the breathing commands required during the abdominal sequences, and communication via the microphone was maintained throughout the scan to ensure safety and comfort, particularly in claustrophobic individuals or those with implants.

### 2.2. MRI Equipment

All MRI examinations were performed at the Smeeni Chronic Disease Hospital (Buzău, Romania) using a 1.5 Tesla scanner (Canon Vantage Orian) with a 16-element body coil (50 cm).

### 2.3. Abdominal Protocol–Sequences and Parameters

For eliminating breathing artifacts, one of the best options is to use the quick sequences provided by the manufacturer. The basic principles of MRI Spin Echo (SE) and Gradient Echo (GRE) sequences have been explained previously [17]. The most used sequences in the abdomen protocol are (1) Single-Shot Fast Spin Echo (Scheme 3), also known as Fast Advanced Spin-Echo (FASE), (2) Steady-State Free Precession (SSFP), and (3) T1-weighted three-dimensional (3D) Gradient Echo (FE), because they can be performed within short breath-hold times [20].

In this study, the different image weighting techniques used for abdominal imaging were as follows:

T2-weighted imaging (T2-WI), typically acquired in the coronal and axial plane, is preferred because it better depicts pathological changes in structures and, when used without fat saturation (Fat Sat), makes it easier to define better the anatomy. When combined with a fat saturation pulse, the subcutaneous fat is hypointense, and fluids appear very bright (CSF or liquid from gallbladder). With an increased TE, fluids maintain their signal, while other structures lose signal and become darker. These sequences are particularly useful for highlighting pathology in regions where fat might obscure lesions or to differentiate between fat and adjacent abnormal tissues, such as tumors or fluid collections.

Diffusion-weighted images (DWI) provide qualitative and quantitative information at a cellular level and are based on T2-weighted imaging, using paired diffusion-sensitizing gradients. In static tissue, these gradients cancel each other out, preserving signal intensity. However, in moving water molecules, rephasing does not occur, leading to signal loss. The greater the water movement, the more signal loss, allowing detection of diffusion. Distinguishing tumors from healthy tissue is possible by detecting differences in water molecule movement. In healthy tissue, water diffuses freely, resulting in higher signal loss. Malignant tumors often restrict water movement, leading to less signal loss and appearing brighter on DWI, making it easier to spot abnormal tissue. However, it is important to note that while diffusion restriction is often observed in malignant tumors, benign lesions, such as hemangiomas, liver abscesses, infected cysts, and hepatic steatosis, may also exhibit mild diffusion restriction. It can be used with different *b* values (s/mm^2^) (*b* = 50, 400, and 800 and in some cases higher) in detection and characterization of a variety of lesions and evaluating tumor response to treatment [21]. When at least two *b* values are used, quantitative analysis can be performed using apparent diffusion coefficient (ADC). ADC values are automatically calculated for all voxels and displayed as a parametric map, enabling the placement of regions of interest (ROIs) for the non-invasive, indirect quantification of tissue cellularity [22].

Jain et al., 2018 [23] demonstrated that the ADC ratio (calculated as the ADC value of the lesion divided by that of the normal liver parenchyma) outperforms absolute ADC values in distinguishing between benign and malignant hepatic lesions. This is because the ratio normalizes inter-patient and scanner-related variability. Their findings showed that an ADC ratio below 0.9 was strongly associated with malignancy, while a ratio above 1.5 indicated a benign etiology. Although DWI and ADC analyses are essential in the evaluation of hepatic lesions, they may not always provide sufficient information for an accurate diagnosis. Therefore, the integration of other MRI sequences is required to complement DWI findings and ensure a comprehensive assessment of liver lesions.

T1-weighted imaging (T1-WI) used in abdominal investigation usually includes T1-WI fat saturation, T1-WI out-of-phase, and in-phase. Imaging with uniform fat suppression is used for multiphase gadolinium-enhanced imaging (arterial and venous) of the upper abdomen and is a critical part of abdominal MRI examinations [7,24]. Dynamic T1-weighted fat saturation imaging is one of the most important sequences for lesion detection and characterization [1]. T1-WI in-phase (IP) and out-of-phase (OOP) are important GE sequences used in abdominal MRI exams, which use the same TR but different TE values. The IP-OOP sequences are mostly used in the study of fat-containing lesions and to identify various disease states related to the presence of fat in the liver (such as diffuse or focal steatosis) [25], by showing a reduction in signal intensities on OOP images compared to IP images. A decrease in signal intensity between in-phase and out-of-phase MR images shows fat.

Water-Fat Separation (WFS-DIXON), as the name suggests, enables fast fat-water separation and also allows us to obtain in-phase and out-of-phase images. In this case, the TEs are set near the time at which the spins of water protons and fat protons are in-phase and out-of-phase, and by calculating the phase difference in the acquired data, the in-phase, out-of-phase, water, and fat images can be reconstructed.

## 3. Examples of Applications of MRI Sequences in Upper Abdominal Analysis

Abdominal MRI can be used in investigating multiple pathologies of the liver, kidneys and renal arteries, adrenal glands, pancreas, gallbladder, and small bowel.

In this section, we detail the MRI protocol used to characterize three hepatic lesions, specifying the sequences and their acquisition order. The examination included the following sequences: T2-weighted imaging (T2-WI), T2-weighted imaging with fat saturation (T2-WI Fat Sat), diffusion-weighted imaging (DWI), in-phase and out-of-phase T1-weighted imaging (T1-WI In/Out of Phase), water/fat separation imaging, T1-weighted imaging before contrast administration (T1-WI Pre-contrast), and dynamic as well as post-contrast T1-weighted imaging (T1-WI Dynamic and Post-contrast).

**Scheme 4:**
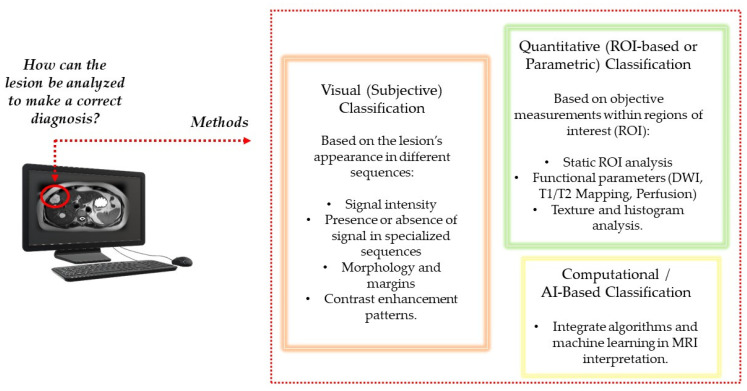
Possible MRI methods for lesion characterization: (1) Visual—based on lesion appearance in different sequences; (2) Quantitative—ROI-based analysis; and (3) AI-Based Methods—integrating algorithms and machine learning in MRI interpretation.

The methods used for lesion characterization traditionally combine visual assessment and quantitative analysis, and in recent years, AI-based approaches (Scheme 4) have also been increasingly employed, allowing for the integration of all three methods [26]. AI-based approaches integrate algorithms and machine learning in the interpretation and utilization of medical images such as MRIs [27].

In this study, we focus on the visual and quantitative methods for lesion characterization.

Initially, based on imaging features, these lesions appear to represent a simple hepatic cyst, a lesion with imaging characteristics suggestive of hemangioma, and an indeterminate lesion of unclear etiology.

Hepatic cysts are common lesions dominated by simple hepatic cysts (well-defined, fluid-filled lesions, with or without an epithelial lining) and are often encountered accidentally, most of them being asymptomatic. Atypical aspects of hepatic cysts should always be carefully examined to avoid misdiagnosing a lesion that requires suitable treatment [28].

Hepatic hemangiomas are the most common benign tumors of the liver, and like the hepatic cyst, they are usually encountered incidentally during imaging investigations for different pathologies. They consist of clusters of blood-filled cavities fed by the hepatic artery and lined by a single layer of flat endothelial cells [29,30].

Hepatic nodular lesions may be either benign or malignant entities, and rigorous imaging evaluation is essential for an accurate diagnostic differentiation.

To differentiate each pathology, sequences with different MR imaging contrasts were used, as each sequence provides specific information for lesion characterization. Certain sequences are acquired prior to contrast administration, while post-contrast sequences are dedicated to evaluating enhancement patterns. Comparison across sequences is important because it allows an accurate diagnosis. Adherence to the recommended sequence order, pre- and post-contrast enhancement, is essential to avoid misinterpretation of lesion characteristics.

The sequences under investigation were independently evaluated on a PACS terminal.

**Figure 1:**
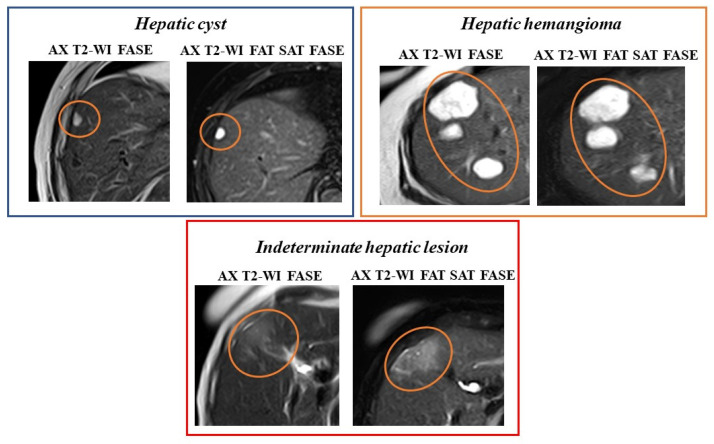
MR imaging comparison of AX T2-WI and AX T2-WI FAT SAT sequences for various lesions: hepatic cyst, hemangioma, and an indeterminate hepatic lesion. The orange circles indicate each lesion’s characteristic signal on the T2-WI sequences.

In Figure 1, on AX T2-WI sequences, both hepatic cysts and hemangiomas appear hyperintense. But certain characteristic differences can be observed between them as follows: the cyst has a well-defined shape and presents a homogeneous signal, while the hemangioma has a more irregular shape and a less homogeneous signal due to fibrous or fatty components. In contrast, the indeterminate hepatic lesion exhibits irregular contours and heterogeneous T2 hyperintensity. On AX T2-WI Fat Sat, fat suppression enhances lesion contrast, improving differentiation of tissue characteristics. Therefore, the T2-WI sequences play an important role in differentiating pathologies based on their characteristics.

**Figure 2:**
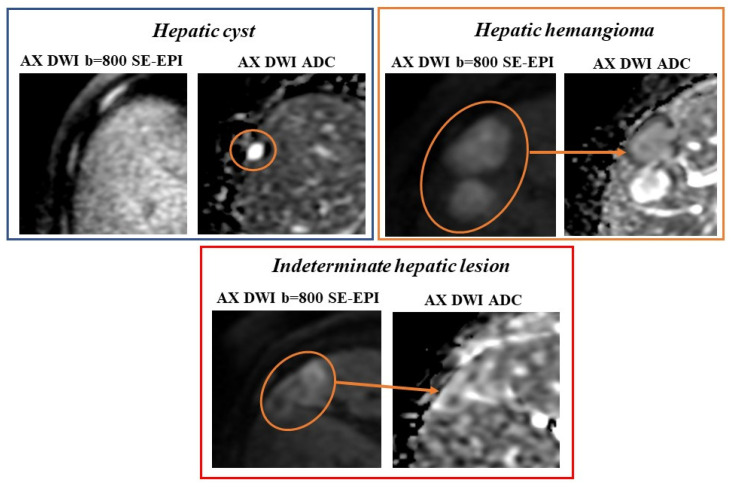
MR imaging comparison of AX DWI SE-EPI and AX DWI ADC sequences for various lesions: hepatic cyst, hemangioma, and an indeterminate hepatic lesion. The orange circles and arrows indicate each lesion’s characteristic signal on DWI (b = 800) and the corresponding finding on the ADC map.

Diffusion-weighted imaging (Figure 2) was acquired with three b-values (50, 400, and 800 s/mm^2^); for illustration and presentation, only the images obtained at b = 800 s/mm^2^ and the ADC map are shown.

It can be observed from Figure 2 that on b = 800 diffusion-weighted images (DWI), the hepatic cyst is not visible, because with the increase in the b-value, the fast diffusion of water leads to a decrease in the signal, so that the cyst becomes hypointense or even invisible. This statement is valid for simple cysts that contain just fluid. Hemangiomas, however, demonstrate mild to moderate diffusion restriction due to their tissue and vascular content, which offers a slight restriction to diffusion. On ADC sequences, the hepatic cysts show high signal intensity due to their fluid content (free diffusion of water), while hemangiomas have intermediate ADC values (slightly limited diffusion of water inside the hemangioma).

The indeterminate hepatic lesion demonstrates important diffusion restriction on both b = 800 DWI and ADC map. As it was shown before, a very important step is to verify the ROI. The ADC values were as follows: cyst ADC = 2.94 × 10^−3^ mm^2^/s, hemangioma = 1.37 × 10^−3^ mm^2^/s, and the indeterminate hepatic lesion ADC = 0.91 × 10^−3^ mm^2^/s.

Using the DWI sequence is important because it is one of the sequences that may help to differentiate between benign and malignant lesions; however, it is important to note that for an accurate diagnosis, it is important to analyze all the sequences.

**Figure 3:**
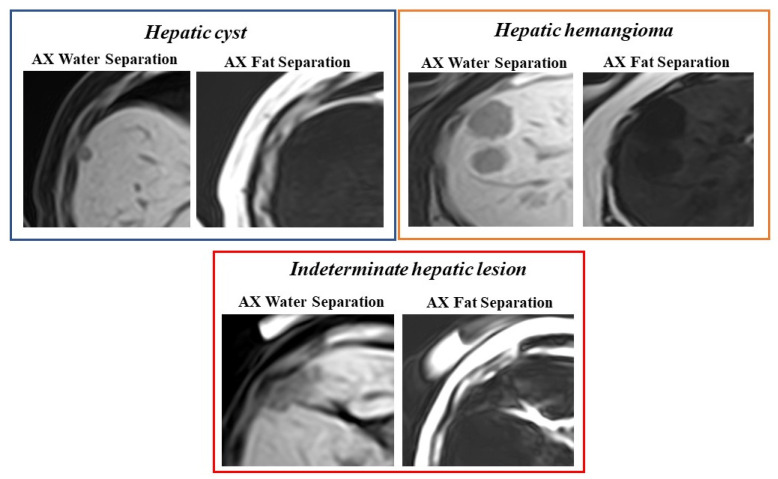
MR imaging comparison of AX water-fat separation sequences for various lesions: hepatic cyst, hemangioma, and an indeterminate hepatic lesion.

In the case of the water-separation sequence (Figure 3), the cyst appears hypointense, indicating that it contains fluid (expected in the case of a simple hepatic cysts), while the hemangioma shows hypointense areas due to blood-filled vascular spaces (typical of hemangiomas). In the case of the indeterminate lesion, mixed signal intensity with some heterogeneity can be seen, indicating possible solid and cystic components. For fat separation (Figure 3), the cyst appears hypointense (dark), confirming the absence of fat content, consistent with a simple fluid-filled lesion. The hemangioma remains relatively hypointense, indicating no fat content, and the indeterminate lesion shows a similar intensity to surrounding tissue, with no convincing evidence of fat content.

**Figure 4:**
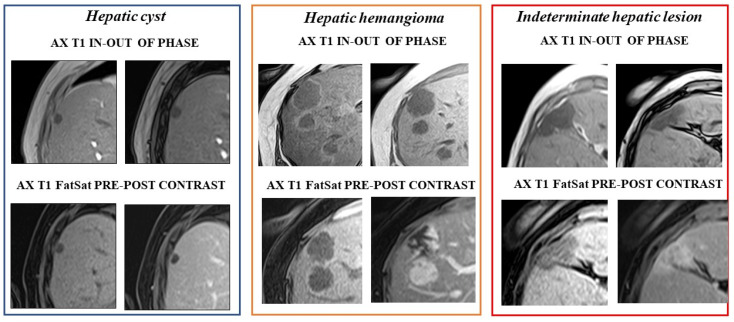
MR imaging comparison of AX T1 in-phase and out-of-phase and AX T1 pre-and post-contrast sequences for various lesions: hepatic cyst, hemangioma, and an indeterminate hepatic lesion.

The in-phase and out-of-phase sequences (Figure 4) show that both the hepatic cyst and the hemangioma exhibit a constant signal without significant changes, indicating the absence of intracellular fat. However, their differentiation becomes evident in the contrast-enhanced sequences, where the hemangioma shows characteristic peripheral enhancement with gradual centripetal filling and progressive contrast enhancement, while the cyst remains non-enhancing, confirming the fluid content. The indeterminate hepatic lesion shows no evidence of intracellular fat on the out-of-phase imaging. On dynamic contrast-enhanced sequences, the lesion demonstrates progressive, predominantly delayed enhancement, more intense than the adjacent liver parenchyma, with a heterogeneous and irregular enhancement pattern suggestive of malignancy, requiring further evaluation.

After analyzing all sequences, the initial diagnosis of simple cyst and hemangioma was confirmed, and the indeterminate hepatic lesion was characterized as possible cholangiocarcinoma.

## 4. Advancing Upper Abdominal Imaging—The Role of MRI Beyond Other Images Techniques

### 4.1. The Use of MRI to Reduce Radiation Exposure in Oncology Patients

This case involves a patient with a history of invasive ductal carcinoma NOS, G2, affecting the left breast, diagnosed at stage II B. The patient underwent chemotherapy and a radical surgical procedure in 2023, followed by radiotherapy and hormone therapy.

Below are the summarized findings from her subsequent imaging investigations:

PET-CT (2 July 2024) investigation shows secondary hepatic lesions and metabolically active nodules. In the case of CT (25 September 2024), the liver was found to be enlarged, containing a stable cyst in segment V. Multiple hypodense nodules were present in the liver with slight peripheral enhancement, showing progression in size and number compared to the CT from June 2024.

**Figure 5:**
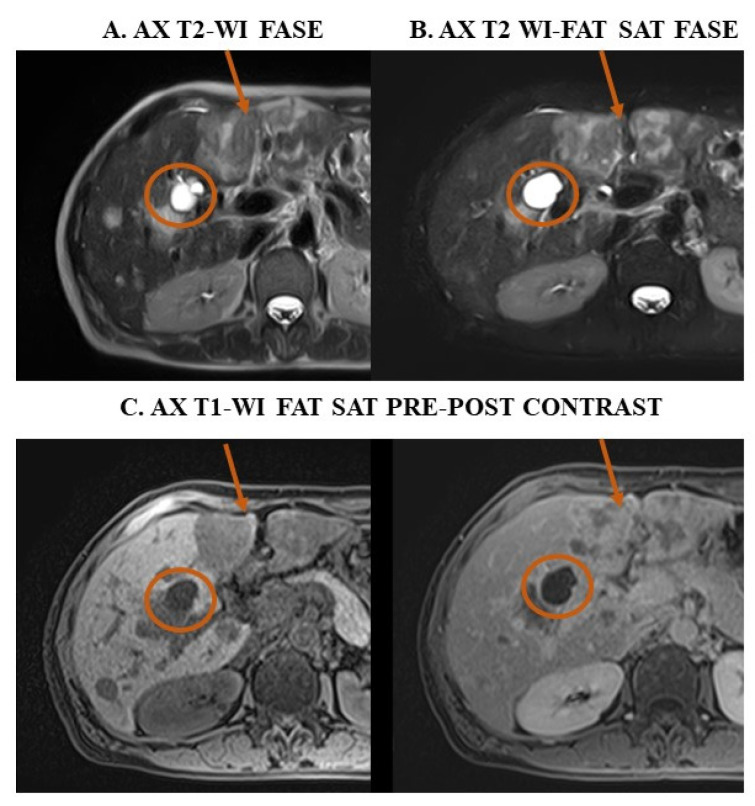
Axial plane of the abdominal view. (**A**) T2-WI, (**B**) T2-WI Fat Sat, and (**C**) T1-WI Fat Sat pre-contrast and post-contrast image. The orange circles and arrows indicate each lesion’s characteristic signal on different sequences, the cyst (orange circle), and hepatic metastasis (orange arrow).

MRI serves as a critical tool in this patient’s management due to its high sensitivity for liver lesions and elimination of additional radiation exposure. Figure 5 shows the differences in contrast and shape between the cyst (orange circle) and hepatic metastasis (orange arrow). Combining MRI with the clinical and laboratory findings ensures a comprehensive, patient-centered approach to care.

### 4.2. Monitoring of Abdominal Lesions from CT to MRI

The patient initially presented in 2022 for the evaluation of a renal lesion. Over three years, serial imaging (CT in 2022, MRI in 2023, and MRI in 2024) was performed for monitoring purposes.

**Figure 6:**
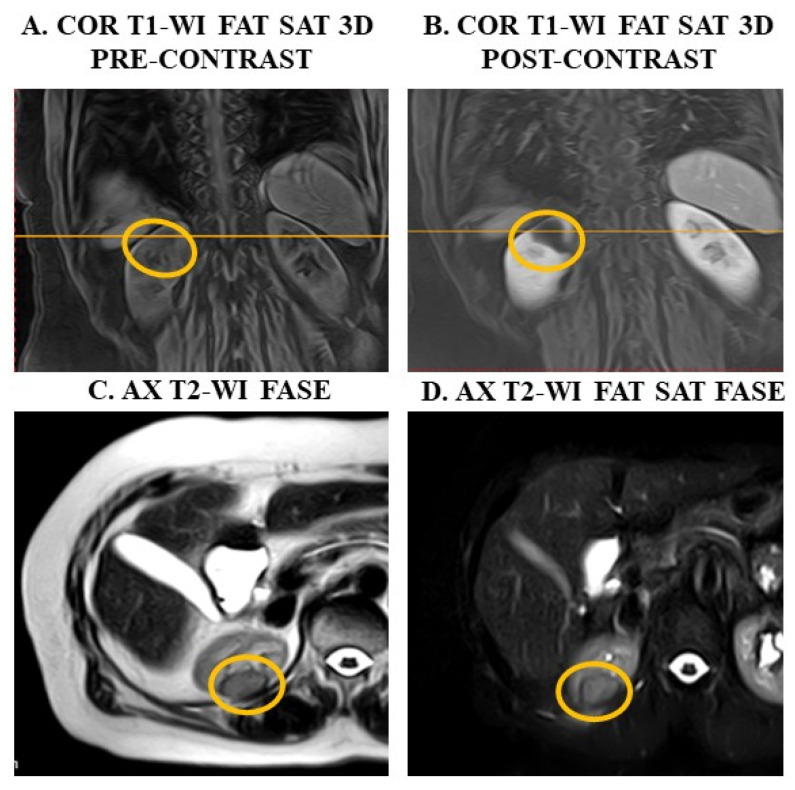
Monitoring of right kidney lesion from 2024 using MRI investigation. The yellow circle highlights the lesion. COR T1-WI Fat Sat image pre-contrast (**A**), post-contrast (**B**). Axial T2-WI FASE (**C**) and AX T2 Fat Sat FASE (**D**).

The initial CT (2022) scan revealed a hyperdense nodule in the upper pole of the right kidney suggestive of a benign lesion (likely Bosniak I). The follow-up MRI (2023) provided a detailed assessment; the size of the right kidney nodule had increased to be classified as Bosniak II, still considered benign but requiring continued surveillance. In the most recent MRI (2024), the right kidney nodule remained relatively stable, retaining its benign characteristics (Bosniak II) (Figure 6). The Bosniak classification may vary depending on the imaging technique used (CT or MRI) due to differences in sensitivity and the criteria applied for each modality [31,32].

The right kidney nodule remained stable for three years. MRI monitoring is important to ensure the stability of the renal lesion and reduce the radiation exposure from CT.

### 4.3. Confirm Upper Abdomen Ultrasound Diagnosis Through MRI

**Figure 7:**
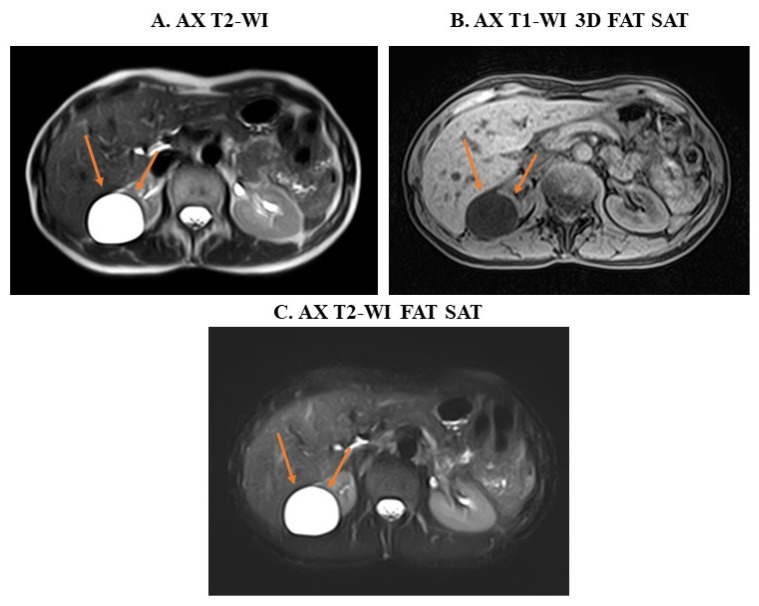
The cystic formation on the right kidney from 2024 MRI investigation. The orange arrow highlights the cyst. (**A**) AX T2-WI, (**B)** AX T1-WI 3D Fat Sat, and (**C**) AX T2-WI Fat Sat.

This case involves a 56-year-old patient who presented with abdominal pain. The ultrasound shows that the right kidney has normal dimensions but showed a cystic formation in the upper caliceal group measuring approximatively 44/40 mm. Because the pain persisted, it was recommended to complete the investigation with MRI. The MRI scan confirmed the presence of the cystic formation on the right kidney, measuring approximatively 46/42 mm (Figure 7) on the right kidney.

In this case, the MRI result confirmed the diagnosis established by the abdominal ultrasound. The diagnostic strategy of ultrasound combined with MRI can sometimes exhibit good performance for upper abdomen. For example, although ultrasound is frequently used as the first-line modality due to its widespread availability and absence of ionizing radiation, its ability to characterize hepatic and pancreatic lesions may be compromised, especially in obese patients, in the presence of overlying bowel gas, or when evaluating small lesions [33]. Also, while ultrasound is operator-dependent and less reproducible, MRI is a standardized and highly reproducible imaging modality. Therefore, combining these techniques could help make a correct diagnosis in such situations.

## 5. Conclusions

In this study, we discuss and show how MRI presents a high interest, being a non-invasive diagnostic method, used as an initial and complementary technique in upper abdominal pathologies. Even though state-of-the-art MRI systems have been implemented and applications have been developed, to deal with pulse sequences, image reconstruction, and data analysis, sequence knowledge plays an important role in personalizing sequences according to the patient’s characteristics (metal, respiratory, movement artifacts).

It was shown how sequences can be optimized using the trade-off parameters and the importance of MRI contrast in differentiation of different pathologies, from a simple cyst to a possible malign tumor. It was also shown that MRI plays an important role as a complementary technique in the investigation of upper abdominal pathologies in order to avoid overexposure to radiation.

The diagnostic strategy involving the use of the two imaging methods, ultrasound and MRI, can present a good performance for the upper abdomen.

## Data Availability

All presented data are available on demand.

## Abbreviations

The following abbreviations are used in this manuscript:

MRI	Magnetic Resonance Imaging
LI-RADS	Liver Imaging Reporting and Data System
SNR	Signal-to-Noise Ratio
NSA/NEX	Number of Averages
DL	Deep learning
TR	Repetition Time
FOV	Field of View
TE	Echo Time
B/W	Receiver Bandwidth
ETL	Echo Train Length
SE	Spin Echo
GE	Gradient Echo
FASE	Fast Advanced Spin-Echo
HASTE	Half-Fourier Acquisition Single-Shot Turbo Spin
SSFSE	Single-Shot Fast Spin Echo
SSFP	Steady-State Free Precession
PSIF	Reverse Fast Imaging with Steady-State Free Precession
T2 FLASH	T2- Fast Low Angle Shot
FE	Field Echo
RF	Radio Frequency
FSE	Fast Spin Echo
IAM	internal auditory meatus
CSF	cerebrospinal fluid
CNR	Contrast-to-Noise Ratio
SE-EPI	Spin Echo—Echo Planer Imaging
AFI	Asymmetric Fourier Imaging
GRASP	Golden-angle RAdial Sparse Parallel
DISCO	DIfferential Subsampling with Cartesian Ordering
T2-WI	T2-weighted imaging
DWI	Diffusion weighted images
ADC	apparent diffusion coefficient
T1-WI	T1-weighted imaging
T1-WI in-phase and out-of-phase	T1-WI IP and OOP
WFS	Water-Fat Separation
Fat Sat	Fat Saturation
CT	Computed Tomography
PET-CT	Positron Emission Tomography—Computed Tomography
